# Successful Management of Primary Gastric Melanoma: A Case Report Emphasizing the Importance of Early Diagnosis and Multidisciplinary Approach

**DOI:** 10.7759/cureus.71673

**Published:** 2024-10-17

**Authors:** Abdelkarim M Barqawi, Alaa Rostom, Duha Suboh, Inam Hindi, Razan Odeh, Hanood Abu Rass

**Affiliations:** 1 Department of General Surgery, An-Najah National University Hospital, Nablus, PSE; 2 Department of Medicine, College of Medicine and Health Sciences, An-Najah National University, Nablus, PSE; 3 Department of Hemato-Oncology, An-Najah National University Hospital, Nablus, PSE; 4 Department of Pathology, An-Najah National University Hospital, Nablus, PSE

**Keywords:** adjuvant immunotherapy, gastrectomy, hemoglobin drop, melanocyte, melanoma

## Abstract

Melanoma is a malignant tumor that develops from the melanocyte-containing epithelial lining of mucosal membranes. This case report highlights a patient who presented with an asymptomatic decrease in hemoglobin (HGB), which necessitated an upper gastrointestinal (GI) endoscopy that identified a necrotic ulcerating lesion on the greater curvature of the stomach, which was confirmed to be a primary malignant gastric melanoma upon biopsy. The multidisciplinary team (MDT) subsequently recommended subtotal gastrectomy with D2 lymphadenectomy. After subsequent surgical intervention, the tumor was successfully removed with clear margins. A successful multidisciplinary approach led to favorable postoperative outcomes, highlighting the importance of timely diagnosis and management of rare presentations of melanoma.

## Introduction

Melanoma is a malignant tumor that develops from the melanocyte-containing epithelial lining of mucosal membranes [1]. It accounts for 1.6%-6.7% of all malignancies and primarily affects adults [2,3]. Cutaneous melanoma is the most common type, accounting for 95% of all other melanomas [2]. Other types have been identified in the eyes and gastrointestinal (GI) organs [2,4]. According to a recent systematic analysis, oropharyngeal melanoma was the most common type, accounting for 32.8% of cases, followed by melanoma of the anal canal (31.4%), rectum (22.2%), esophagus (5.9%), stomach (2.7%), small bowel (2.3%), gallbladder (1.4%), and large bowel (0.9%) [5].

In many cases, the primary cutaneous melanoma metastasizes to the gastrointestinal tract. However, in 1%-4% of the diagnostic workup of patients with cutaneous primary melanoma and up to 60% of melanoma patients at autopsy, a metastatic melanoma to the gastrointestinal tract is usually found [2]. However, in a few cases, it originates primarily from the gastrointestinal mucosa [4,6,7]. Primary gastric melanoma (PGM) is a very rare entity for which fewer than 50 cases have been reported [8]. Its clinical manifestations are not specific and mimic those of gastric adenocarcinoma or lymphoma [3], so it is frequently misdiagnosed as many other malignant gastric tumors [4].

The gold standard for a mucosal melanoma diagnosis is histology and microscopic visualization of melanin [1]. Moreover, CT, MRI, and digital radiography could be helpful in diagnosis [4].

There are no known major risk factors for the occurrence of PGM [1]. Research suggests that PGM arises from the melanoblastic cells of the neural crest, which migrate to the ileum through the omphalomesenteric canal, or from amine precursor uptake and decarboxylation (APUD) cells, which undergo neoplastic transformation [2].

Here, we described a case of PGM to increase awareness of uncommon tumors and improve the general understanding for better tumor diagnosis and treatment.

## Case presentation

A 50-year-old male patient with a free past medical history presented to our hospital complaining of generalized fatigue and facial pallor and denied any history of other symptoms, such as nausea, vomiting, or changes in bowel habitus. The patient underwent investigations, including a complete blood count (CBC), and his hemoglobin (HGB) level was 9.3 g/dL. Iron therapy and multivitamin supplementation were given with close follow-up.

As part of his follow-up, a repeated CBC revealed an HGB of 7.3 mg/dL. As a result, he was admitted to another hospital urgently for further evaluation. An investigation revealed a positive fecal occult blood test, so he was referred to our hospital for upper and lower gastrointestinal endoscopy.

**Figure 1:**
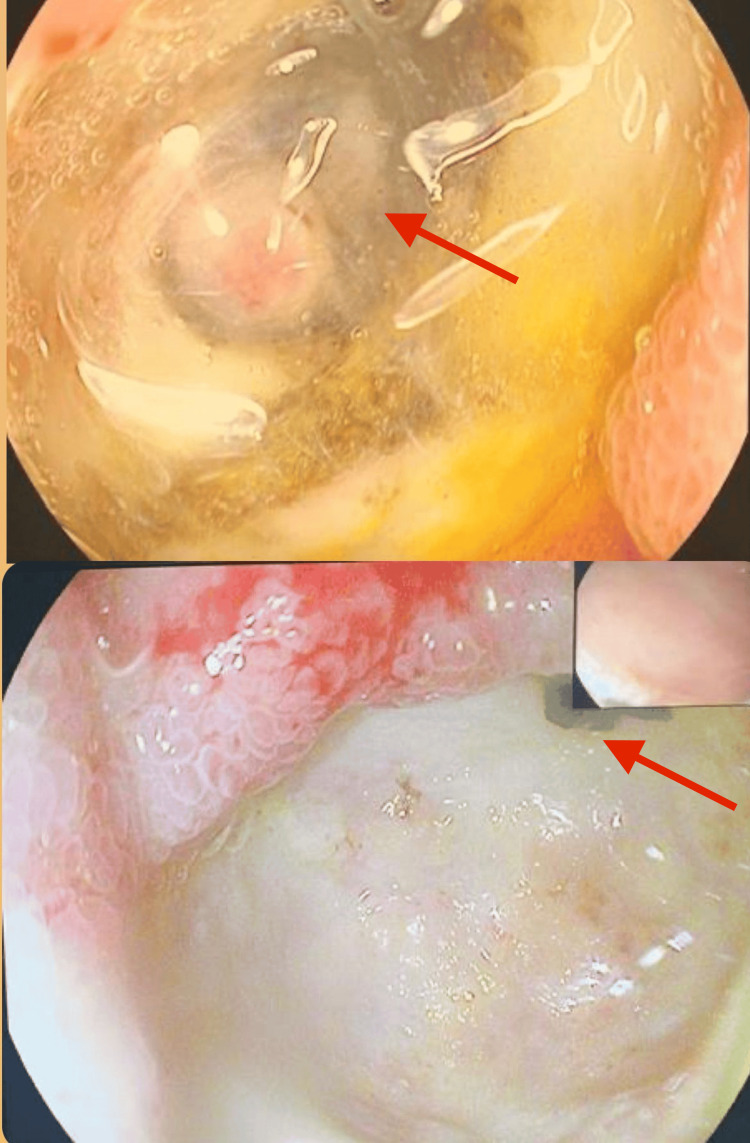
Endoscopic view Fungating lesion occupying the proximal body with a necrotic ulcerating center on the greater curvature side (arrow).

Diagnostic upper gastrointestinal endoscopy revealed a fungating lesion occupying the proximal body with a necrotic ulcerating center on the greater curvature side (Figure 1).

A biopsy sample was taken, which later indicated malignant melanoma.

**Figure 2:**
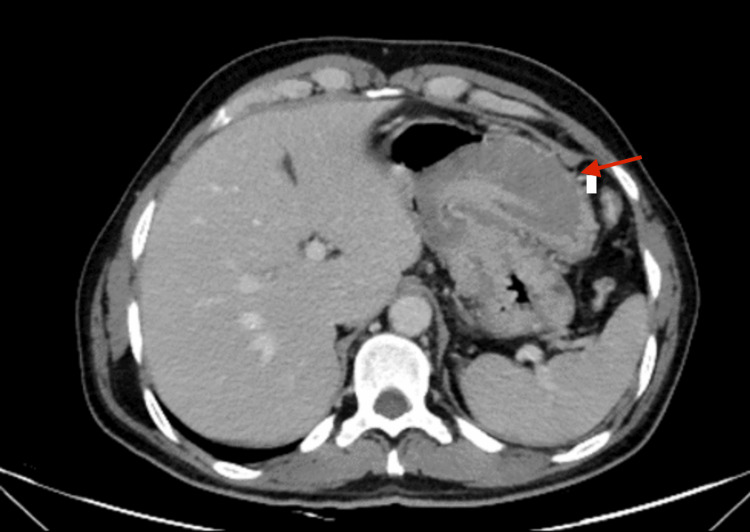
Abdominal CT scan Gastric wall thickening at the proximal greater curvature (arrow). CT: computed tomography

Staging chest, abdomen, and pelvis CT scans were performed and showed gastric wall thickening at the proximal greater curvature (Figure 2).

Additionally, a PET-CT scan revealed non-metastatic disease with uptake only in the parotid gland in addition to the malignant gastric ulcer.

After a multidisciplinary team (MDT) discussion, the patient was scheduled for subtotal gastrectomy with D2 lymphadenectomy. One week before the scheduled surgery, the patient developed frequent episodes of melena associated with dizziness and exertional shortness of breath, as well as symptomatic tachycardia and hypotension. His hemoglobin was 4.5 mg/dL, so he was urgently admitted to the intensive care unit (ICU) for upper gastrointestinal bleeding.

An urgent endoscopy revealed a bleeding tumor. Blood transfusion was started, and intravenous proton pump inhibitors (PPIs) were administered. After stabilization, the patient was transferred to the operating theater, D2 subtotal gastrectomy with Roux-en-Y gastrojejunostomy was performed, with a safety margin of 6 cm proximally, and the distal resection margin included the first 2 cm of the duodenal bulb.

**Figure 3:**
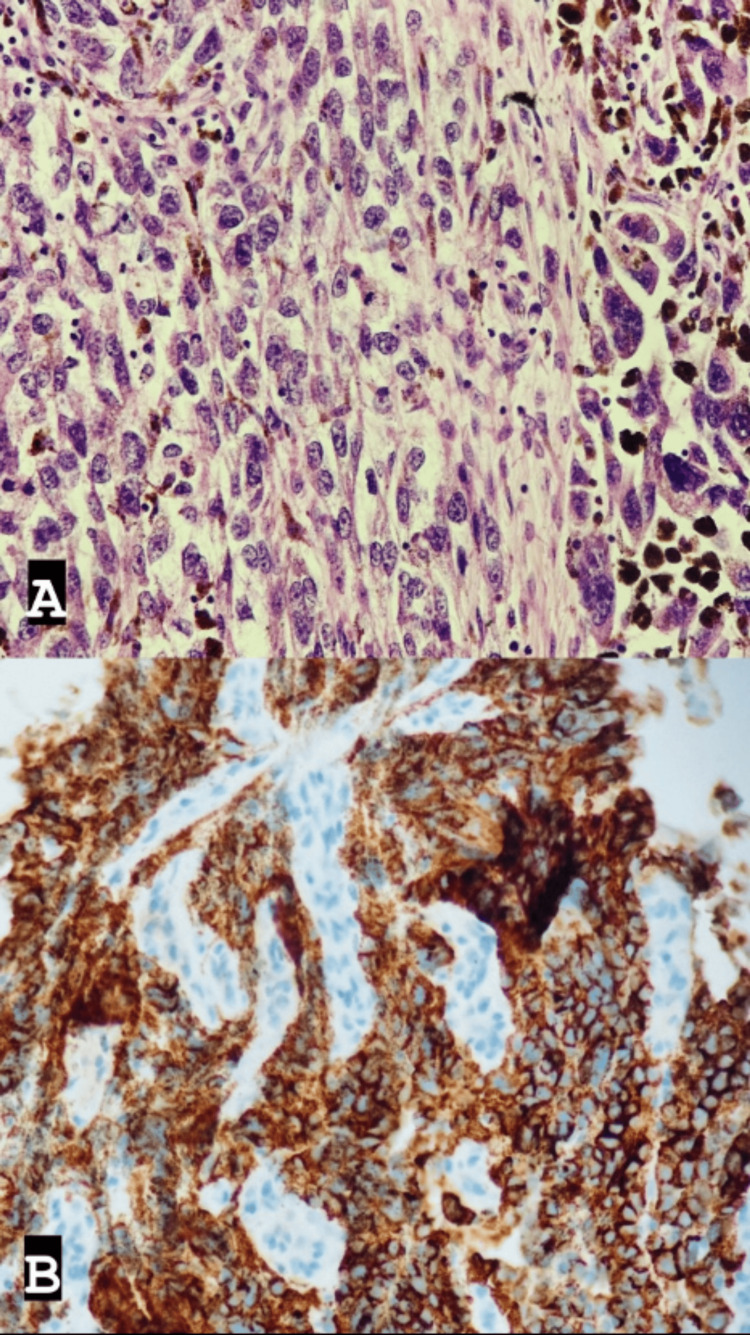
Histopathology slides (A) Proliferation of spindled melanocytes with prominent nucleoli and melanin pigmentation. (B) The tumor cells show diffuse expression of HMB-45 immunostain. HMB-45: human melanoma black-45

Histopathological findings of the resected specimen revealed a histological type of malignant melanoma in which the proximal and distal margins were free of tumor, the tumor invaded the muscularis propria, no subserosal extension was noted, and no lymphovascular or perineural invasion was observed in 0/12 lymph nodes (LNs) (Figure 3).

The postoperative course was unremarkable, and after one week, the patient was discharged home. Oncology discussion after histopathology revealed that the patient had high-risk features, and he needed adjuvant immunotherapy for one year; therefore, he was started on adjuvant pembrolizumab.

## Discussion

PGM is a rare malignant tumor that must satisfy a specific criterion to be diagnosed [2]. These criteria include the absence of a primary melanoma at another site and characteristic histopathological findings such as in situ changes in the overlying or adjacent GI epithelium. Furthermore, disease-free survival of at least 12 months following curative surgical excision of the involved organ is considered one of the criteria to distinguish PGM from metastatic, as 50% of patients with stage IV melanoma or visceral disease from an unknown primary tumor typically die within 12 months of diagnosis [2], emphasizing the necessity for early detection [1].

Primary gastric melanoma can closely mimic other gastric cancers or more benign conditions. The patient's initial presentation of iron deficiency anemia (IDA) underscores the importance of considering malignancy in unexplained anemia patients [1]. The progression from initial anemia to gastrointestinal bleeding highlights the aggressive nature of PGM and the necessity for timely diagnosis and intervention [9].

Endoscopic and imaging studies are crucial for identifying the tumor and planning the surgical approach, as in our patient, gastric wall thickening and a tumor with a necrotic center on the greater curvature were observed, similar to previously reported studies [1]. Moreover, the most common location for PGM is the body of the stomach, followed by the cardia, antrum, and fundus, as stated in previously published research.

Regarding PGM management, no conventional treatment has been suggested due to the rarity of the disease. The most commonly used method for treatment is surgical resection, which is still widely recommended. Surgical procedures vary as the size and location of tumors vary; however, in most cases, the range of resection should not be smaller than that for stomach cancer.

Previous systematic studies including 25 patients revealed that, with the exception of one patient, all of them underwent open surgical resection of the tumor. Depending on the size and location of the tumor, surgical excision ranged from total gastrectomy to partial gastrectomy. Depending on the degree of lymphatic dissemination [5].

Our patient was similarly managed with partial gastrectomy and lymphadenectomy. Adjuvant chemotherapy and immunotherapy are also commonly used, especially PD-1 inhibitors (toripalimab) combined with paclitaxel [6,10]. The postoperative course went smoothly for this patient. Some previous studies reported postoperative fever, wound infection, small bowel ischemia, respiratory complications, and death [5]. Notably, patients who underwent surgical resection without subsequent chemotherapy frequently developed metastases and died, while those treated with immunotherapy sometimes experienced tumor bleeding, necessitating discontinuation of treatment [6].

## Conclusions

PGM is a rare malignant tumor that must satisfy a specific criterion to be diagnosed and differentiated from secondary melanoma and can resemble any gastric tumor at presentation, so a careful diagnosis and MDT between oncology, general surgery, and pathology are mandatory for better evaluation and management.
